# Lysosomal acid lipase deficiency: A rare inherited dyslipidemia but potential ubiquitous factor in the development of atherosclerosis and fatty liver disease

**DOI:** 10.3389/fgene.2022.1013266

**Published:** 2022-09-20

**Authors:** Katrina J. Besler, Valentin Blanchard, Gordon A. Francis

**Affiliations:** Department of Medicine, Centre for Heart Lung Innovation, Providence Research, St. Paul’s Hospital, University of British Columbia, Vancouver, Canada

**Keywords:** lysosomal acid lipase, LIPA, Wolman Disease, Cholesteryl Ester Storage Disease, GWAS, smooth muscle cells, atherosclerosis, nonalcoholic fatty liver disease

## Abstract

Lysosomal acid lipase (LAL), encoded by the gene *LIPA*, is the sole neutral lipid hydrolase in lysosomes, responsible for cleavage of cholesteryl esters and triglycerides into their component parts. Inherited forms of complete (Wolman Disease, WD) or partial LAL deficiency (cholesteryl ester storage disease, CESD) are fortunately rare. Recently, LAL has been identified as a cardiovascular risk gene in genome-wide association studies, though the directionality of risk conferred remains controversial. It has also been proposed that the low expression and activity of LAL in arterial smooth muscle cells (SMCs) that occurs inherently in nature is a likely determinant of the propensity of SMCs to form the majority of foam cells in atherosclerotic plaque. LAL also likely plays a potential role in fatty liver disease. This review highlights the nature of LAL gene mutations in WD and CESD, the association of LAL with prediction of cardiovascular risk from genome-wide association studies, the importance of relative LAL deficiency in SMC foam cells, and the need to further interrogate the pathophysiological impact and cell type-specific role of enhancing LAL activity as a novel treatment strategy to reduce the development and induce the regression of ischemic cardiovascular disease and fatty liver.

## Introduction

Lysosomal acid lipase (LAL), encoded by the gene *LIPA*, is the sole lysosomal lipase, responsible for the critical functions of hydrolysis of cholesteryl esters (CE) contained in endocytosed lipoproteins to component free cholesterol and fatty acids, and of triglycerides to their component fatty acids. LAL also cleaves stored CE delivered to lysosomes for breakdown in the lipophagy component of autophagy (Ouimet et al., 2011), enhancement of which may be protective against atherosclerosis (Sergin et al., 2017). Complete deficiency of LAL, Wolman Disease (WD), is fatal in early life due to malabsorption and liver disease (Grabowski et al., 2019). Near total LAL deficiency or cholesteryl ester storage disease (CESD) can have a variable phenotype but is frequently asymptomatic and difficult to identify clinically despite affected individuals having only as little as 1–12% of residual LAL activity (Bernstein et al., 2013). Recombinant LAL is now available and lifesaving for individuals with WD and useful to prevent and reverse liver fat accumulation and fibrosis and correct the dyslipidemia in CESD. In recent years *LIPA* has been identified as a cardiovascular risk allele, though the directionality of this association is not yet clear. More recently, expression of *LIPA*/LAL has been found to be low in arterial smooth muscle cells of both humans and mice, as a natural occurrence rather than due to mutations, and is the apparent cause of CE overload in lysosomes of smooth muscle cell foam cells in atherosclerosis. Low blood LAL activity has also been found to be associated with nonalcoholic fatty liver disease. In this review we summarize recent knowledge about the nature of LAL, mutations in *LIPA* leading to WD and CESD, the value of *LIPA* as a predictor of risk for atherosclerosis, and recent findings regarding its role in atherosclerosis and fatty liver disease.

## *LIPA*/LAL regulation and function

The *LIPA* gene is located on chromosome 10q23.2–23.3 (Anderson et al., 1993) and encodes a 372 amino acid polypeptide with a 27 amino acid signal sequence necessary for both secretion and lysosomal targeting of LAL (Ameis et al., 1994). Purification of human LAL from liver tissue yields two glycoproteins of 56 kDa and 41 kDa, thought to represent a proprotein and a mature, active protein respectively (Ameis et al., 1994). The propeptide sequence, cleavage site, and location and identity of the putative responsible protease have not been identified. It has recently been suggested that LAL is not in fact a proprotein and that the 41 kDa form observed is reflective of protease cleavage during purification (Strøm et al., 2020). Mature LAL is glycosylated in the endoplasmic reticulum (ER), and mannose-6-phosphate is added in the Golgi, a critical step for lysosomal targeting and for cellular uptake of secreted or exogenous LAL by receptor-mediated endocytosis (Sando and Henke, 1982; Du et al., 1998; Zschenker et al., 2005). The pathway of trafficking and secretion of LAL, and molecular characterization of key sequences and modifications of LAL remain to be further elucidated; discussion of these remaining questions is further described in a recent review (Li and Zhang, 2019).

Structurally, LAL is similar to gastric and lingual lipase, notably containing a core domain with a catalytic triad of Ser153, Asp324, and His355 (Lohse et al., 1997), an oxyanion hole and a cap domain containing a lid which regulates substrate entry (Roussel et al., 1999; Rajamohan et al., 2020). This family of lipases is active at acidic pH, with optimal pH for LAL being 3.5–4.5 (Dubland and Francis, 2015), and is not homologous with neutral lipases, such as hormone sensitive lipase and neutral cholesteryl ester hydrolase (Anderson and Sando, 1991). Recent elucidation of the crystal structure of LAL suggests that this is mediated by protonation of Asp-361 at acidic pH, allowing opening of the lid (Rajamohan et al., 2020).

CE and triglycerides are the major substrates of LAL, and upon hydrolysis, free cholesterol and free fatty acid products are released from lysosomes. Free cholesterol and free fatty acids subsequently inhibit SREBP activation in the endoplasmic reticulum, thereby reducing new cholesterol synthesis and low density lipoprotein receptor expression, and free cholesterol can be converted to oxysterol metabolites that activate LXRα to promote expression of genes including ATP-binding cassette transporter AI (ABCA1) and ABCG1 that promote cholesterol efflux from cells (Venkateswaran et al., 2000; Horton et al., 2002). LAL therefore plays a critical role in regulation of cellular lipid metabolism in response to cellular lipid accumulation by inhibiting *de novo* cholesterol synthesis and LDL uptake, and by promoting removal of excess cholesterol from cells (Goldstein et al., 1975; Brown et al., 1976; Bowden et al., 2011). LAL is the sole neutral lipid hydrolase in the lysosome and thus is critical for hydrolysis of CE as well as triglycerides contained in endocytosed lipoproteins, and subsequent cholesterol efflux (Goldstein et al., 1975). Due to promoting ABCA1 expression and mediating release of free cholesterol from endocytosed lipoproteins (Bowden et al., 2011), LAL is a key driver of total body reverse cholesterol transport (Bowden et al., 2018). Injection of LAL^−/−^ mouse macrophages containing LDL radiolabeled with ^3^H-CE into LAL^+/+^ mice resulted in significantly higher appearance of ^3^H-cholesterol in feces compared to the same injection of lipoproteins into LAL^−/−^ mice. This also demonstrated the ability of supplemental LAL to be taken up by cells and correct reverse cholesterol transport *in vivo* (Bowden et al., 2018).

LAL is similarly necessary for metabolism of lipids contained in apoptotic cells endocytosed by macrophages during efferocytosis (Viaud et al., 2018). In lipoprotein-loaded macrophages, LAL is also essential for metabolism of CE contained in cytosolic lipid droplets via lipophagy, a selective process of lipid degradation where lipid droplets are fused with lysosomes (Ouimet et al., 2011). LAL expression is also activated in conditions of nutrient deprivation by transcription factors forkhead homeobox type protein O1 (FOXO1) in adipocytes (Lettieri Barbato et al., 2013) and transcription factor EB (TFEB) in hepatocytes and other cell types (Settembre et al., 2013), as part of an autophagic and specifically lipophagic response, to mobilize fatty acids and free cholesterol. LAL also plays critical roles in lipid metabolism beyond autophagy, such as supplying free fatty acids by catabolism of triglycerides that drives alternative activation of M2 macrophages (Huang et al., 2014). During monocyte to macrophage differentiation, LAL is upregulated by Sp1 and AP-2, possibly to accommodate increased lysosomal degradation processes in macrophages (Ries et al., 1998a). Plaque macrophages may therefore contribute to increased LAL activity in the atherosclerotic relative to normal artery wall (Dubland and Francis, 2015).

Epigenetic regulation of LIPA has been reported in a number of recent studies. Preconception maternal exposure to saturated fatty acids is associated with increased *LIPA* DNA methylation in infants (Robinson et al., 2020), and similarly, children of mothers with Type 1 Diabetes Mellitus exhibit hypermethylation and increased expression of *LIPA* (Knorr et al., 2021. Preprint.). The *LIPA* promoter is hypomethylated in obese patients with elevated LDL (Płatek et al., 2020), and *LIPA* is hypermethylated in patients with alcohol dependence (Brückmann, 2019. Dissertation.). Correlation of DNA methylation data with RNA and protein expression, and specific investigation of *LIPA* regulation and contributions to these phenotypes, will be necessary to determine the significance of these findings. DNA methylation master regulator UHRF1 and DNA methyltransferase inhibitor zebularine may upregulate *LIPA* in mesothelioma cells and mouse melanoma, respectively (Fang et al., 2021; Reardon et al., 2021), and histone deacetylase inhibitor voronistat increases LAL protein and activity in fibroblasts (Subramanian et al., 2017). Further characterization of the epigenetic regulation of *LIPA* and possible epigenetic modulators have potential for developing novel regulators of LAL expression.

LAL is present in all cell types with the exception of erythrocytes (Grabowski et al., 2019), and participates in multiple physiological processes in different tissues. Additional roles for LAL have been elucidated in thermogenesis (Duta-Mare et al., 2018; Fischer et al., 2021; Fischer et al., 2022), bile acid metabolism with effects on the gut microbiome (Sachdev et al., 2021), insulin sensitivity (Radović et al., 2016), and retinoic acid metabolism (Grumet et al., 2016).

## LAL deficiency states–Wolman Disease and CESD

### Mutations

**FIGURE 1:**
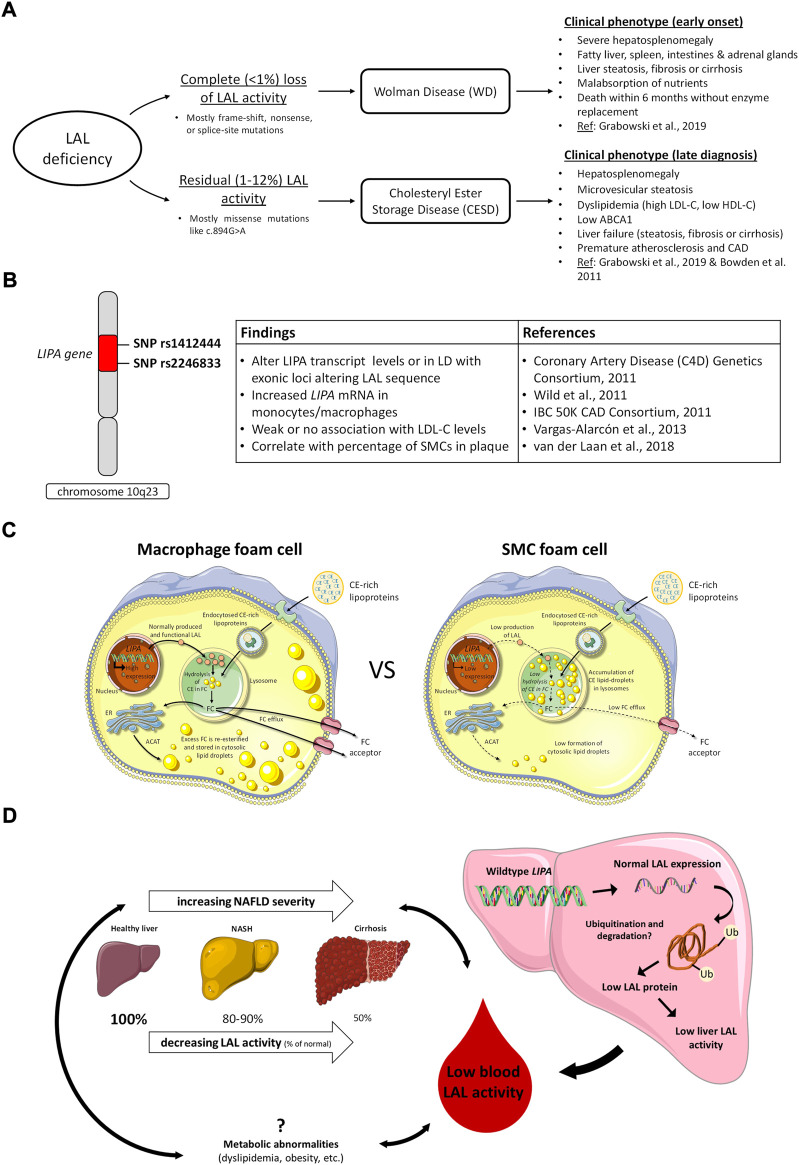
LAL in deficiency states, GWAS studies of cardiovascular risk variants, and potentially common roles of relative LAL deficiency in the development of atherosclerosis and nonalcoholic fatty liver disease. **(A)**, clinical consequences of complete and partial LAL deficiency; **(B)**, *LIPA* variants identified in GWAS studies of CVD risk and related effects in cells and tissues. LD, linkage disequilibrium; **(C)**, variable expression of *LIPA*/LAL in macrophages and smooth muscle cells (SMCs) and consequences for cell cholesterol handling. In macrophages, high levels of *LIPA*/LAL expression result in efficient hydrolysis of endocytosed lipoprotein cholesteryl esters (CE) to free cholesterol (FC), and trafficking of lysosomally-released FC for re-esterification in the endoplasmic reticulum (ER) or removal from the cell by cholesterol efflux mechanisms. In SMCs, low levels of LIPA/LAL expression result in retention of lipoprotein CE in lysosomes, reducing FC available for re-esterification in the ER or efflux from cells. Adapted from (Dubland et al., 2021); **(D)**, Potential role of LAL in nonalcoholic fatty liver disease (NAFLD). LAL activity in the blood is reduced in NAFLD and lower LAL activity is associated with increased NAFLD severity. Both NAFLD and low LAL are associated with metabolic abnormalities, but no causal relationships between these factors have been determined. No variants in *LIPA* have been associated with NAFLD, and expression of LAL in NAFLD livers is normal, but activity is low (Gomaraschi et al., 2019), possibly due to accumulation of dysfunctional LAL (Carotti et al., 2021).

Mutations in *LIPA* leading to reduced LAL activity present as two rare disorders, Wolman Disease (WD) and Cholesteryl Ester Storage Disease (CESD). They are referred to collectively as LAL deficiency (LALD). WD involves complete loss or less than 1% preservation of LAL activity (Aslanidis et al., 1996; Pagani et al., 1998; Porto, 2014), and was first described in 1956 by Wolman *et al.* who found cholesterol-laden foam cells throughout the liver, spleen, intestine, and adrenal glands of an infant female presenting with vomiting and severe hepatosplenomegaly (Abramov et al., 1956). Infants with WD typically present before 4 months of age with gastrointestinal symptoms and a reduced growth rate, and death follows in the first 6 months to 1 year of life from liver failure and malnutrition without treatment (Marshall et al., 1969; Grabowski et al., 2019). Patients with CESD, conversely, typically retain between 1–12% of LAL activity (Bernstein et al., 2013; Porto, 2014) and exhibit a range of less severe phenotypes which often remain unrecognized until later childhood or into adulthood (Bernstein et al., 2013; Aguisanda et al., 2017; Rashu et al., 2020) (Figure 1A). CESD patients characteristically will exhibit hepatomegaly, elevated serum transaminases, and dyslipidemia including reduced high density lipoprotein cholesterol (HDL-C), due to impaired upregulation of ABCA1 in response to CE sequestration in lysosomes (Bowden et al., 2011), and elevated low density lipoprotein cholesterol (LDL-C) (Bernstein et al., 2013). The progressive liver pathology caused by CESD, which involves hepatocyte accumulation of LAL substrates in the lysosome as well as lipid-overloaded liver macrophages and fibrosis (Hůlková and Elleder, 2012; Grabowski et al., 2019), may be diagnosed as non-alcoholic fatty liver disease, non-alcoholic steatohepatitis, or cryptogenic liver disease (Bernstein et al., 2013). Often CESD patients are also at risk for accelerated atherosclerosis and premature cardiovascular disease, thought to be due to persistent dyslipidemia (Bernstein et al., 2013; Burton et al., 2015b). Additional reduction of naturally lower LAL activity in arterial smooth muscle cells (SMCs) compared to macrophages may also further promote SMC foam cell formation in atherosclerosis (see below) (Dubland et al., 2021). The most common cause of premature death in CESD patients is liver failure, which may occur in childhood, younger or older adulthood depending on the severity of LAL deficiency (Bernstein et al., 2013).

At the cellular level, LALD causes the accumulation of lipoprotein-derived CE particularly, and triglycerides, in the lysosome. This leads to a perceived cellular deficiency of both free fatty acids and free cholesterol, which cannot or are only minimally released from the lysosome due to the reduced activity of LAL. Free fatty acids and free cholesterol normally interact with SREBP (1c and 2) causing feedback inhibition of HMG-CoA reductase which reduces cholesterol synthesis, LDL receptor downregulation to reduce LDL uptake, acyl-cholesterol acyltransferase activation to esterify free cholesterol, and downregulation of fatty acid synthesis (Horton et al., 2002; Reiner et al., 2014). In LALD, the reverse occurs: in addition to increased apolipoprotein B and very low density lipoprotein (VLDL) production (Reiner et al., 2014), lack of free cholesterol released from the lysosome also impairs ABCA1 upregulation and cholesterol efflux to apolipoprotein A-I, leading to low HDL production and impaired whole body reverse cholesterol transport (Bowden et al., 2011; Bowden et al., 2018). This interruption of intracellular cholesterol flux results in excessive lipid accumulation and dysfunction in multiple cell types, manifesting most prominently in the liver in CESD and in multiple tissues in WD.

WD and CESD are both autosomal recessive disorders, but in most cases are caused by distinct mutations in the *LIPA* gene, and can be distinguished biochemically by the level of residual LAL activity. CESD is therefore *not* the heterozygous form of WD, but is less severe due to the nature of the *LIPA* mutations present in both alleles. WD mutations are more often frame-shift, nonsense, or splice-site mutations rather than missense mutations, which are more likely to result in CESD (Vinje et al., 2018). The most common CESD-causing variant is a splice site mutation, c.894G>A, which allows production of a small percentage of functional *LIPA* transcripts, whereas WD splice-site mutations result in a total lack of functional LAL transcripts (Aslanidis et al., 1996; Pagani et al., 1998). Similarly, degradation of LAL protein secondary to misfolding due to deletions or other mutations results in zero functional protein with WD mutations and some functional protein with CESD mutations (Fasano et al., 2012). Large structural changes in LAL or changes in the critical amino acids of the catalytic triad or other key regions also typically manifest as WD, whereas smaller structural changes are more likely to manifest as CESD (Saito et al., 2012). It must be considered that compound heterozygosity, where a patient has two different mutations in their two copies of *LIPA*, is common (Ries et al., 1998b; Santillán-Hernández et al., 2015; Cappuccio et al., 2019). This can manifest as patients having one copy of a WD-type mutation but produce some functional LAL from the other copy with a CESD mutation, and therefore present with CESD rather than WD (Lohse et al., 2000).

### Prevalence

In 2007, about half of previously reported cases of CESD were c.894G>A (also known as E8SJM) carriers (Muntoni et al., 2007); the frequency of c.894G>A is therefore often used to estimate prevalence of CESD. The same 2007 study showed a 1 in 200 carrier frequency of c.894G>A in a German population, and estimated a 1 in 40,000 prevalence of CESD, whereas a recent meta-analysis by Carter *et al.* estimates a carrier frequency of 1 in 336 and CESD prevalence of 1 in 160,000 (Carter et al., 2019). It is evident that LALD frequency differs depending on ethnicity and geographical location: a New York multiethnic cohort had c.894G>A carrier frequencies of 1 in 1,000 in Asian populations, 1 in 333 for Caucasian and Hispanic, and no African American carriers (Scott et al., 2013). There are limitations to using c.894G>A for estimates of prevalence: this mutation is not present in WD patients (Grabowski et al., 2019), and the proportion of LALD cases carrying this mutation may vary in different populations. Carter *et al.*, by interrogating an international genomic database for multiple *LIPA* mutations, estimated an alternate general population LALD prevalence of 1 in 177,452 (Carter et al., 2019), while a newer study combining functional data and 165 published LALD genotypes with multiple public genomic databases estimated a prevalence between 1 in 170,000 and 290,000 (del Angel et al., 2019). It is clear that LALD is a very rare disease, with differing prevalence in diverse populations, but high carrier frequencies and presence in multiple ethnic groups suggesting potential benefit for routine screening.

### Screening and diagnosis

Several recent studies screening patients with hypercholesterolemia or elevated transaminases using the dried blood spot assay (DBS) for blood LAL activity (Hamilton et al., 2012) have failed to identify cases of LALD (Pullinger et al., 2015; Sjouke et al., 2016; Ashfield-Watt et al., 2019): one Norwegian study screened 3,000 hypercholesterolemic patients without an autosomal dominant cause of hypercholesterolemia (Vinje et al., 2018), while a United Kingdom study screened 1825 patients with both low HDL-C and elevated transaminases (Reynolds et al., 2018), with no cases of LALD discovered. The majority of patients in these studies were adults. One study screening for LALD based on clinical suspicion (hepatomegaly, a 1.5-fold increase in transaminases compared to reference limits, or dyslipidemia with or without splenomegaly, gastrointestinal dysfunction, or liver steatosis, fibrosis, or cirrhosis) successfully identified 19 cases in 4,174 using DBS (Tebani et al., 2021). Another study screening 810 children in Turkey with either elevated transaminases for 3 months, hepatomegaly, or liver steatosis, fibrosis, or cirrhosis not explained by obesity or other causes by DBS found two LALD patients (Kuloglu et al., 2019). A recent study published in this series of Frontiers in Genetics screened 669 Slovenian children with familial hypercholesterolemia and detected 3 cases of LALD homozygous for c.894G>A (Sustar et al., 2022). Despite the rarity of LALD, with many cases undiagnosed until later in life, and the availability of effective treatment, screening especially in children with hypercholesterolemia or liver disease after eliminating common causes may be beneficial (Vinje et al., 2018; Carter et al., 2019).

Diagnosis of LALD relies on measurement of LAL activity via the DBS assay. Patients with dyslipidemia, including elevated LDL and low HDL, who on taking a family history do not exhibit an autosomal dominant pattern of inheritance that would otherwise be consistent with familial hypercholesterolemia, can be examined for possibly hepatomegaly and/or splenomegaly and tested for elevated ALT. If this combination of abnormalities is present, increased liver echogenicity on ultrasound, cirrhosis or steatosis of the liver, and early cardiovascular disease, this is suggestive of LALD and LAL DBS testing should be performed. Deficient or slight residual LAL activity is diagnostic for LALD. Patients with marginal LAL activity can then be diagnosed if *LIPA* gene sequencing reveals a pathogenic variant. The reader may refer to diagnostic flowcharts published by Reiner et al. and Lipiński et al. (Reiner et al., 2014; Lipiński et al., 2018).

Although LALD is considered an autosomal recessive disease, indicating a lack of disease phenotype in heterozygous carriers, it is possible that mild phenotypes or increased risk may result from reduced LAL activity in these individuals. There is one report of carriers of c.894G>A showing elevated total serum cholesterol (Muntoni et al., 2013), and a higher prevalence of this mutation was found in familial hypercholesterolemia cohorts than in the general population in two studies (Sjouke et al., 2016; Ashfield-Watt et al., 2019); another study showed no association of c.894G>A with serum cholesterol (Stitziel et al., 2013). Heterozygous carriers of LALD mutations do not always have LAL activity falling below reference levels by DBS (Lukacs et al., 2017); it is possible that these individuals would have no phenotype. Carriers of multiple different CESD and WD mutations with no disease but between 30–70% of normal LAL activity by DBS were also shown to have altered macrophage differentiation (Rothe et al., 1997). Broader screening approaches for LALD may identify more carriers and allow further study of possible heterozygous phenotypes. We discuss below associations of cardiovascular disease risk with more common *LIPA* variants and functional correlates; note that these differ from known disease-causing mutations in *LIPA*.

### Treatment

Prior to 2015, the only treatment available for LALD was supportive. Dietary restriction of fats with nutrient supplementation to prevent malabsorption in WD cannot extend survival beyond approximately 1 year of age, and lipid-lowering medications such as statins do not correct liver disease in CESD (Erwin, 2017). Liver transplantation for WD or CESD is limited by availability and by allograft rejection, and often ends in disease recurrence, possibly due to failure to correct LAL deficiency in bone-marrow derived macrophage-monocytes which infiltrate multiple tissues (Bernstein et al., 2018). Hematopoietic stem cell transplant (HSCT) for WD is limited by complications and comorbidities and rarely extends survival beyond 1 year (Jones et al., 2016; Erwin, 2017). In 2015, an enzyme replacement therapy for LAL was approved. Sebelipase alfa is a recombinant human LAL protein, administered intravenously every one to 2 weeks (CADTH Common Drug Review, 2018). It is typically dosed at 1 mg/kg for CESD and 3–5 mg/kg for WD patients (Jones et al., 2017; Demaret et al., 2021; Burton et al., 2022). Cellular uptake of sebelipase alfa occurs via the mannose receptor, which then facilitates delivery to the lysosome (Balwani et al., 2013). At treatment initiation, sebelipase alfa in CESD patients can lead to transient increases in blood triglycerides, total cholesterol, and LDL-C (Balwani et al., 2013; Burton et al., 2015a), thought to be caused by mobilization of free cholesterol and free fatty acids with increased LAL activity. After 7–20 weeks of treatment, and continuing up to 5 years after treatment initiation, triglycerides, total cholesterol, and LDL-C are lowered and HDL-C is increased. Both short term and long term reduction of serum transaminases, liver fat content, and liver volume also occur with sebelipase alfa treatment (Balwani et al., 2013; Valayannopoulos et al., 2014; Burton et al., 2015a; Malinová et al., 2020; Burton et al., 2022). Key characteristics and major findings from each sebelipase alfa trial are outlined in Supplementary Table S1.

In WD, similar improvements in lipid and liver function parameters were observed with sebelipase alfa treatment in two studies, with trends in another study compared to baseline (Jones et al., 2017; Demaret et al., 2021; Vijay et al., 2021); the smaller difference between pre and post-treatment values in the latter study may have occurred due to very early treatment initiation (median 7 weeks of age). Relief of symptoms including nausea and diarrhea, resolution of hepatosplenomegaly, and increased weight for age were also shown with treatment, as well as reduced requirement for nutritional support. Survival in the first 12 months increased from 11% in a historical untreated control population to 67–100% with sebelipase alfa, with 68% surviving to 5 years of age and one patient surviving 10 years (Demaret et al., 2021; Vijay et al., 2021) (Supplementary Table S1). Earlier initiation of treatment when patients are in more stable condition appears to improve outcome. Follow-up with surviving patients continues, and long-term effects of treatment remain to be studied.

Sebelipase alfa has been shown to be safe in both short and long-term studies, for both CESD and WD patients. Mild infusion reactions occur in most patients, with infrequent severe hypersensitivity-like responses that can be resolved with diphenhydramine or epinephrine (Balwani et al., 2013; Burton et al., 2015a; Demaret et al., 2021; Vijay et al., 2021; Burton et al., 2022). In almost all cases, reducing the dose then slowly returning to the initial dose is effective and patients are able to continue treatment even after severe reactions. Anti-drug antibody (ADA) formation occurs in some CESD patients and most WD patients (Balwani et al., 2013; Jones et al., 2017; Demaret et al., 2021; Vijay et al., 2021; Burton et al., 2022) (Supplementary Table S1). This is likely due to the lack of endogenous LAL present in WD compared to CESD. ADA do not appear to correlate with adverse reactions in either WD or CESD, however in WD, patients with ADA may show reduced response to treatment (Vijay et al., 2021), whereas this was not observed in CESD (Burton et al., 2015a; Burton et al., 2022). The issue of reduced response to treatment due to ADA in WD patients may be addressed by combining enzyme replacement therapy with hematopoietic stem cell transplant (HSCT). A small study treating WD patients with sebelipase alfa conducted subsequent HSCT in patients who exhibited reduced response or ADA, and reported good outcomes (Potter et al., 2021). This approach may improve outcomes of HSCT, since patients are more stable upon initiation, and circumvent problems with continuous central venous access and ADA in treating WD with sebelipase alfa, since successful HSCT could provide longer-term correction of LAL deficiency (Stein et al., 2007; Potter et al., 2021).

Parents of children with LALD report feelings of uncertainty and powerlessness upon wrestling with a new diagnosis of LALD (Hassall et al., 2022). Sebelipase alfa has provided incredible increases in survival, reduction of disease in terms of lipid and liver parameters, and a good safety profile. Limitations remain however, such as infusion reactions and ADA, problems with continuous central venous access in WD patients (Demaret et al., 2021), the frequency of dosing, and the cost of treatment. Effects of treatment begin to reverse after a few weeks without sebelipase alfa such that biweekly doses are required (Balwani et al., 2013), and sebelipase alfa can cost between $892 000 and $4.9 million CDN annually per patient (CADTH Common Drug Review, 2018). Gene therapies using mRNA to promote LAL expression or gene-editing techniques to correct monogenic defects for sustained effect may be an approach to reduce costs, frequency of dosing, and ADA. Continued follow-up will also be necessary to determine longer term response to treatment.

### LALD and atherosclerosis

Premature atherosclerosis has often been reported in CESD. This has not been observed in WD, perhaps due to the short lifespan of these patients (Grabowski et al., 2019). Sebelipase alfa reduces LDL-C, LDL particle number, and apolipoprotein B, and increases HDL-C and apolipoprotein A1, suggesting an anti-atherogenic effect (Wilson et al., 2018). It is unknown whether sebelipase alfa has additional effects on atherosclerosis risk outside of its modulation of lipid parameters, and larger and longer term studies would be necessary to determine cardiovascular event rates. Interestingly though, it appears that sebelipase alfa improves lipid parameters with or without previous treatment with lipid-lowering medication (LLM), and that LLMs have an additive effect with sebelipase alfa in patients who started LLMs during the trial period (Valayannopoulos et al., 2014; Wilson et al., 2018; Burton et al., 2022). These effects require confirmation in more patients with appropriate study designs. Whether enzyme replacement therapy in individuals who do not have a genetic deficiency in LAL might modulate atherosclerosis risk is a separate question, which we address below.

## Identification of *LIPA* as a cardiovascular risk allele in GWAS studies

Genome-wide association studies (GWAS) of large European, South Asian and Mexican cohorts have identified *LIPA* single nucleotide polymorphisms (SNPs) rs1412444 and rs2246833 on chromosome 10q23 as common variants associated with coronary artery disease (CAD) risk (Coronary Artery Disease (C4D) Genetics Consortium, 2011; IBC 50K CAD Consortium, 2011; Wild et al., 2011; Vargas-Alarcón et al., 2013). While these SNPs are intronic and therefore not directly coding LAL sequence, the risk SNPs are proposed to alter *LIPA* transcript levels by affecting its rate of transcription, nuclear export and transcript stability, or to be in linkage disequilibrium with exonic loci altering LAL sequence. Several studies have linked these risk alleles to increased expression of *LIPA* mRNA by circulating monocytes (Coronary Artery Disease (C4D) Genetics Consortium, 2011; Wild et al., 2011; PLOS Genet 2011) but not to changes in LDL-C level (Figure 1B). No clear association has been found with *LIPA* expression in the liver. These findings suggest enhanced *LIPA* expression may not affect the CE hydrolytic activity of LAL but have some other effect on CAD risk, possibly related to endothelial dysfunction as measured by reduced flow-mediated dilatation (Wild et al., 2011). In contrast to CESD, where very low LAL activity results in elevated LDL-C, low HDL-C levels and increased risk of atherosclerosis (Dubland and Francis, 2015), CAD risk variants have generally not been found to be associated with alterations in plasma lipid levels. Potential hypotheses for increased CAD risk with increased *LIPA* transcript levels could be increased extracellular release of LAL-generated free fatty acids having a pro-inflammatory effect in the plaque intima, increased hydrolysis of CE by exocytosed LAL on LDL retained in the interstitium leading to increased interstitial free cholesterol promoting plaque inflammation, and increased LAL-modified LDL uptake and foam cell formation by intimal macrophages and SMCs.

*In vitro* studies to determine the effect of *LIPA* risk variants on LAL function and activity and cell cholesterol metabolism have reached differing conclusions. Morris et al. investigated a coding variant rs1051338, which is in high linkage disequilibrium (*r* ^2^ = 0.89) with the GWAS variant rs2246833 and causes a nonsynonymous threonine to proline change within the signal peptide of LAL (Morris et al., 2017). COS7 cells transfected with the risk allele exhibited significantly less LAL protein and activity, felt to be due to an increased rate of LAL protein degradation. These results were confirmed in lysosomal extracts of macrophages from 4 individuals homozygous for either the nonrisk or risk allele of rs1051338, in which inhibition of the proteasome resulted in equal amounts of lysosomal LAL protein in risk and nonrisk macrophages (Morris et al., 2017). While this effect of reduced LAL activity would be consistent with the pro-atherogenic effect of LAL deficiency in CESD, potential weaknesses of this study are that rs1051338 is not yet proven as the causal variant at the *LIPA* GWAS locus, the small sample size, and that other aspects of LAL activity such as CE hydrolysis and autophagy were not examined (Zhang and Reilly, 2017). Evans et al. also studied the effect of rs1051338 variant in human monocytes from a larger patient cohort, n = 114, and found that it conferred increased *LIPA* expression and LAL activity, but had no effect on *LIPA* mRNA or LAL activity or secretion when transfected into HEK-293T or NIH-3T3 cells (Evans et al., 2019). Their conclusion was that common *LIPA* exonic variants in the signal peptide are of minimal functional significance and that CAD risk is instead associated somehow with increased *LIPA* function linked to intronic variants (Evans et al., 2019). The effect of *LIPA* variants identified by GWAS studies therefore remains controversial, and will require further studies to identify the role of variant expression in other relevant cell types such as SMCs, and in targeted mouse models with knock in of human *LIPA* risk and non-risk alleles to assess the effect on atherosclerosis (Zhang and Reilly, 2017). Interestingly, a study by van der Laan *et al.* using carotid plaque specimens from two independent biobanks identified expression of *LIPA* variant rs1412444 correlated most strongly with percentage of SMCs in plaque, and predicted reduced CAD risk with the GWAS SNP (van der Laan et al., 2018). If SMCs, like macrophages, have higher LAL activity with this SNP, that would imply a protective effect of increasing LAL in plaque SMCs.

## Low expression of LAL by arterial SMCs and formation of SMC foam cells in atherosclerosis

Expression of *LIPA*/LAL varies considerably among tissues (Du et al., 1996). As early as 1974 de Duve proposed that low LAL might be contributing to accumulation of CE in aortic cells in atherosclerosis (de Duve, 1974). Subsequent reports indicated low levels of *LIPA* expression in mouse (Du et al., 1996) and human (Zhang et al., 2017) SMCs relative to macrophages and other tissues, but the functional significance of that in atherosclerosis was not explored. We had previously reported that low LAL-mediated hydrolysis of lipoprotein CE is the reason for low ABCA1 expression in human CESD fibroblasts, is the likely reason for low plasma HDL-C in CESD, and that supplementation of human CESD fibroblasts with exogenous LAL corrects ABCA1 expression and activity (Bowden et al., 2011). We had also reported that ABCA1 expression is low in intimal compared to medial arterial SMCs (Choi et al., 2009), and in coronary artery intima SMCs compared to intima macrophages (Allahverdian et al., 2014). We subsequently quantitated the contribution of SMCs to total atheroma foam cells, and by immunohistochemical analysis determined that, at minimum, SMCs contribute >50% of total foam cells in human coronary atheromas (Allahverdian et al., 2014). We subsequently found that, using gentle digestion of atheromas and flow cytometry, that SMCs contribute approximately 70% of foam cells in apoE-deficient mice fed a Western diet for 6 weeks, in both SMC nonlineage-tracing and lineage-tracing mice, and that SMC foam cells in mice also exhibit low ABCA1 expression relative to macrophage foam cells (Wang et al., 2019). Defective cholesterol handling by arterial SMCs is therefore a likely major cause of cholesterol accumulation in atherosclerotic plaque.

In an attempt to determine the mechanism of reduced ABCA1 expression by SMCs, we determined that SMCs exhibit reduced production of 27-hydoxycholesterol and fail to activate cholesterol esterification and inhibit *de novo* cholesterol synthesis when compared to human macrophages in response to loading with aggregated LDL (Dubland et al., 2021). These findings suggested SMCs are deficient in trafficking of lysosomally-derived cholesterol following lipoprotein loading. Strikingly, we observed sequestration of lipoprotein-derived CE within the lysosomes of SMCs even after a 24 h equilibration period following aggregated LDL loading; macrophages on the other hand sequester cholesterol within cytosolic CE droplets following hydrolysis of lipoprotein CE and trafficking of lysosomally-derived free cholesterol to the endoplasmic reticulum for re-esterification (Dubland et al., 2021). This suggested either that SMCs have impaired lysosomal function or that deficiency of *LIPA*/LAL may explain this striking phenotype. Similar to previous results from Jerome and others (Griffin et al., 2005; Cox et al., 2007), we determined that loading of human monocyte-derived macrophages with aggregated LDL led to free cholesterol accumulation and reduced acidification in lysosomes of macrophages, but we found no similar defects, nor any defect in proteolysis, in SMC lysosomes (Dubland et al., 2021). We then determined that SMCs have markedly low *LIPA* mRNA, LAL protein and LAL activity both before and after loading the cells with aggregated LDL (Dubland et al., 2021) (Figure 1C). These *in vitro* studies were confirmed in human coronary atheromas, showing high LAL expression in macrophages but low LAL in arterial SMCs, particularly intimal SMCs (Dubland et al., 2021); these results were also corroborated in apoE-deficient mouse atheroma where macrophages showed high and arterial SMCs showed low *LIPA* expression (Dubland et al., 2021). While additional reasons for low ABCA1 in human SMCs were determined, including lower expression of sterol-27-hydroxylase and LXRα, necessary components of enhanced ABCA1 expression, incubation of human SMCs with exogenous LAL significantly increased lysosomal CE hydrolysis and cholesterol efflux to apolipoprotein A-I using the cells’ existing level of ABCA1 expression (Dubland et al., 2021). This suggests that increasing *LIPA* expression and/or LAL activity in arterial SMCs may be effective in promoting removal of excess cholesterol from SMC foam cells in plaque atheroma, assuming sufficient apolipoprotein A-I and HDL acceptor particles are available for both ABCA1-dependent and–independent cholesterol efflux, respectively. Precedent for this possibility is provided by a pre-clinical study of Hong Du and others where weekly injection of yeast-derived LAL for 6 weeks both prevented appearance and induced regression of atheromas in LDL receptor-deficient mice; however, the cellular target of the exogenous LAL in those studies was unknown (Du et al., 2004). Based on plaque macrophages having abundant LAL expression (Ries et al., 1998a; Dubland et al., 2021) and not showing increased cholesterol efflux in response to LAL supplementation (Dubland et al., 2021), the results suggest plaque SMC foam cells were the target of the exogenous LAL. It also provided highly suggestive evidence that increased circulating LAL can have a major therapeutic effect upon diffusing into the artery wall.

## Mechanism of reduced *LIPA* expression in arterial SMCs

The reason for low *LIPA*/LAL expression by arterial SMCs relative to macrophages is not yet known. SMCs are not designed or equipped to carry out the functions of professional macrophages. Even though plaque SMCs can express multiple macrophage markers (Allahverdian et al., 2014; Feil et al., 2014; Shankman et al., 2015), induction of macrophage protein expression by cholesterol loading SMCs in culture was accompanied by only ∼25% of the ability of those SMCs to carry out phagocytosis and efferocytosis when compared to cultured macrophages (Vengrenyuk et al., 2015). The concept of a “SMC-derived macrophage” is therefore likely incorrect: even when expressing macrophage markers, dedifferentiated SMCs retain a distinct SMC-specific gene expression pattern and do not assume the capabilities of professional macrophages (Vengrenyuk et al., 2015). SMCs are however critical for the initial deposition of lipoproteins in the human artery wall, through secretion of negatively-charged proteoglycans that bind and retain positively-charged apolipoprotein B-containing lipoproteins like LDL in the intima (Hurt-Camejo and Camejo, 2018; Allahverdian et al., 2022). These retained lipoproteins surround primarily SMCs rather than macrophages in the deep intima in human atherosclerosis-prone arteries (Nakashima et al., 2007), and through expression of scavenger receptors (Allahverdian et al., 2012), though of less abundance than in macrophages, over time can be taken up to generate SMC foam cells. This capacity of SMCs to take up retained lipoproteins to become foam cells, but not have the same capacity as macrophages to catabolize lipoprotein CE due to low *LIPA* expression, may be a consequence of human evolution not progressing as rapidly as the increase in atherosclerosis as a cause of death in the last 100 years. When humans didn’t live as long due to death from famine, infection or war*, i.e.*, did not live long enough to die from atherosclerosis, the inability of SMCs to catabolize lipoproteins as well as macrophages had no major consequences. Now with lifespans averaging over 80 years in many developed countries, this lack of adequate SMC LAL activity has a functional consequence. This strongly indicates the need to further investigate the pathophysiologic impact of enhancing LAL activity in SMC foam cells in the artery wall.

The possibility also exists that low *LIPA*/LAL expression represents a defect in the regulation of this gene in arterial SMCs. A generalized defect in lysosomal function in SMCs seems unlikely based on their ability to carry out lysosomal proteolysis normally (Dubland et al., 2021), and to apparently carry out autophagy normally (Salabei and Hill, 2013). A recent publication suggested SMC foam cells isolated from mouse atheromas have a higher level of dysfunctional autophagy during atherogenesis relative to macrophage foam cells (Robichaud et al., 2022). Whether upregulating known inducers of *LIPA* expression such as FOXO1 or TFEB can rescue *LIPA* expression and LAL function in SMC foam cells remains to be determined. TFEB overexpression has been shown to increase *LIPA* expression and lysosomal biogenesis in mouse peritoneal macrophages (Emanuel et al., 2014), and to reduce atherosclerosis in a mouse model (Sergin et al., 2017); whether the significantly lower *LIPA* expression in SMCs could be similarly enhanced requires further investigation.

## The role of LAL in fatty liver disease

It is established that genetic LALD causes a microvesicular steatosis and fatty liver disease, but a role for LAL in the more common cases of non-alcoholic fatty liver disease (NAFLD) found in the general population is now being investigated. NAFLD is characterized by fat accumulation or steatosis in the liver, and has a worldwide prevalence of approximately 25% (Friedman et al., 2018). The beginning stages of steatosis do not carry a high morbidity, but insidious progression to non-alcoholic steatohepatitis (NASH), fibrosis, and finally liver cirrhosis leads to liver failure and other adverse outcomes, including hepatocellular carcinoma (Friedman et al., 2018). The most common cause of death in patients with NAFLD is cardiovascular disease, probably related to a combination of both the metabolic abnormalities (diabetes, obesity, dyslipidemia, etc.) associated with both NAFLD and cardiovascular disease, and hepatic inflammation independently promoting systemic vascular damage and coagulation (Tana et al., 2019). The progression of NAFLD is often asymptomatic and may not be reflected in laboratory values. Diagnosis of NAFLD via non-invasive imaging modalities is reliable, but NASH diagnosis requires liver biopsy (Friedman et al., 2018). Blood LAL activity by DBS has been shown to be lower in NAFLD than in healthy subjects in multiple studies (Baratta et al., 2015a; Thoen et al., 2021): one key study by Baratta *et al.* shows LAL activity is inversely correlated to severity of NAFLD, with lower activity in NAFLD non-NASH than healthy patients (∼10% reduction), even lower in NASH patients (∼20%), and further reduced in patients with cirrhosis (∼50%) (Baratta et al., 2019a). Lower LAL activity in NASH is associated with increased necroinflammation and NASH severity (Vespasiani-Gentilucci et al., 2017). Some studies did not detect differences in earlier stages of NAFLD, but LAL by DBS was found to be reduced in cirrhosis of both cryptogenic (Vespasiani-Gentilucci et al., 2016; Gravito-Soares et al., 2019) and known etiologies (Vespasiani-Gentilucci et al., 2016; Ferri et al., 2020). Gravito-Soares *et al.* showed that LAL activity can be used to predict cryptogenic cirrhosis and fibrosis more effectively than existing markers (Gravito-Soares et al., 2019). LAL is therefore a very attractive biomarker for NAFLD, because the DBS assay is non-invasive, can be incorporated as part of regular bloodwork, and appears to predict progression of NAFLD with reasonable sensitivity and specificity (Gravito-Soares et al., 2019). Continued study will be necessary to verify the utility of LAL as a biomarker of NAFLD, and it remains to be seen whether LAL activity could be used to identify patients earlier in the NAFLD progression than existing methods.

It should be noted that there are limitations to the DBS assay for LAL activity. DBS assays testing for other lysosomal storage diseases mostly reflect leukocyte enzyme activity, and since activity is normalized not to protein content or cell counts but to blood spot area, blood leukocyte count could importantly influence results (Civallero et al., 2006; Ceci et al., 2011). Platelets also have lysosomes and thus contribute part of the activity measured by DBS (Bentfeld-Barker and Bainton, 1982). WBC and platelet counts have both been reported to be increased in NAFLD steatosis and decreased in cirrhosis, so these factors are key to consider in interpreting DBS results (Qamar and Grace, 2009; Chao et al., 2022). A study investigating platelet and leukocyte influence on DBS results in healthy subjects showed a greater correlation of total DBS LAL activity with LAL activity in platelets than in leukocytes, and observed an association of total activity with platelet but not leukocyte count (Vespasiani-Gentilucci et al., 2017). Several of the studies showing low LAL activity in NAFLD also found that LAL activity was associated with leukocyte count (Baratta et al., 2019b) or both leukocyte and platelet counts (Vespasiani-Gentilucci et al., 2016; Tovoli et al., 2017), but in all of these LAL activity was independently associated with NAFLD after adjusting for leukocytes and platelets.

The pathogenesis of the lowered LAL activity in NAFLD is as yet unknown. Multiple studies screening for LAL mutations in NAFLD patients with low LAL activity did not find any (Vespasiani-Gentilucci et al., 2016; Gravito-Soares et al., 2019), and there are currently no GWAS reporting associations of NAFLD or other hepatic phenotypes with *LIPA* variants in the NHGRI-EBI GWAS catalog (Buniello et al., 2019), so it does not appear to be a gene polymorphism effect. An interesting study by Carotti *et al.* suggests a post-translational mechanism, demonstrated using *in vitro* and mouse models, and confirmed in NAFLD patient biopsies, that NAFLD is associated with low functional levels of LAL protein and accumulation of dysfunctional ubiquitinated LAL (Carotti et al., 2021). In agreement with this, Gomaraschi *et al.* report that low LAL activity in liver biopsies of NAFLD patients was shown to be independent of LAL expression (Gomaraschi et al., 2019). Other etiologies of fatty liver disease also exhibit lowered LAL activity, including alcoholic and HCV-related liver disease, though NAFLD shows a greater reduction of LAL (Angelico et al., 2017; Tovoli et al., 2017; Ferri et al., 2020). This suggests that lowered LAL activity is partly secondary to liver disease, since various etiologies share the same characteristic of low LAL, but the even lower LAL in NAFLD specifically suggests that the metabolic abnormalities associated with NAFLD may also be related to LAL activity directly (Baratta et al., 2015b; Angelico et al., 2017). We have discussed above the association of low LAL with dyslipidemia; Baratta *et al.* show an association of elevated LDL with low LAL activity in NAFLD patients (Baratta et al., 2015a). Thoen *et al.* similarly associate low LAL with high BMI in NAFLD patients, and *LIPA* variants have previously been associated with metabolic syndrome, though this association requires further investigation (Guénard et al., 2012; Thoen et al., 2021). Thoen *et al.* also demonstrate increased necroinflammation in NASH patients with lower LAL activity, which may speak to the immune roles of LAL (Thoen et al., 2021). It is unclear from these associations what the relationship between low LAL, metabolic or inflammatory factors associated with NAFLD, and NAFLD liver pathology is: low LAL may be secondary to both NAFLD and the identified factors, causative of both, or somewhere in between (Figure 1D).

Baratta *et al.* speculate that determining the epigenetic or metabolic factors leading to low LAL may be instrumental in treating NAFLD, and suggest that sebelipase alfa enzyme-replacement therapy may be useful in these patients (Baratta et al., 2015b). Sebelipase alfa has not yet been tested for NAFLD, CAD, or any indication other than LALD. Hepatic overexpression of LAL using lentivirus in a mouse model of NAFLD led to reduced steatosis and fibrosis, decreased liver inflammation, and decreased serum lipid levels (Li et al., 2021); however, overexpression with an adeno-associated virus in a different mouse model of NAFLD did not attenuate steatosis, and increased liver inflammation (Lopresti et al., 2021). Both studies showed increased autophagic activity in hepatocytes. *In vitro,* a novel GATA3-binding molecule morroniside increases *LIPA* expression and reduces the fibrosis response in hepatic stellate cells (An et al., 2022). Further testing in alternate animal models for NAFLD, or using different strategies to increase hepatic LAL, will be necessary to elucidate the utility of increasing LAL as a treatment for NAFLD. It is possible that some models, and some patients, with lower endogenous LAL activity may respond better to such treatment than those with normal LAL activity.

## Conclusion

LAL is critical to life as the sole neutral lipid hydrolase in lysosomes, as a director of downstream cholesterol metabolism in cells, and as a driver of total body reverse cholesterol transport. Total deficiency of LAL is fatal, whereas even very low levels of residual LAL activity are compatible with life though predisposing to liver disease and potentially atherosclerosis. In addition to utilizing recombinant LAL to treat complete and partial LAL deficiency, we are now learning much more about the role of *LIPA* expression as a predictor of cardiovascular risk, and the likelihood that naturally occurring low levels of *LIPA* expression predict the formation of SMC foam cells, the proposed primary source of foam cells in atherosclerosis. It is likely that in the next decade the activity and functions of LAL will assume an ever increasing role in our understanding of the pathogenesis and potential treatment of both atherosclerosis and fatty liver disease.
