# Supplementary material for: Lysosomal acid lipase deficiency: A rare inherited dyslipidemia but potential ubiquitous factor in the development of atherosclerosis and fatty liver disease
Source: Front Genet. 2022 Sep 20;13:1013266. doi: 10.3389/fgene.2022.1013266 (PMC9530988; doi:10.3389/fgene.2022.1013266)
Supplement: Supplementary file 1 [file Table1.DOCX]

**Supplementary Table 1. Summary of Sebelipase Alfa trials in LALD patients.**

Abbreviations: CESD, cholesteryl ester storage disease; LAL, lysosomal acid lipase; ADA, anti-drug antibodies; TC, total cholesterol; LDL, low density lipoprotein cholesterol; HDL, high density lipoprotein cholesterol; TG, triglycerides; IAR, infusion-associated reaction; apo, apolipoprotein; ALT, alanine aminotransferase; DBS, dried blood spot; PBMC, peripheral blood mononuclear cells.

| Patient characteristics | Number of participants | Dose and treatment period | Baseline LAL activity* | Study conclusions | Citation |
| --- | --- | --- | --- | --- | --- |
| CESD  Age 19-45 | 9 | 0.35, 1, or 3 mg/kg weekly for 4 weeks | 35+/- 15 umol/g/h in leukocytes (central lab lower limit of normal is 350) | - Well-tolerated - No ADA - Transaminases decreased, many to normal levels - TC, LDL, and TG first increased then decreased - Transaminases increased off-treatment | (Balwani et al., 2013) CL-01 |
|  | 7 | 0.35, 1, or 3 mg/kg weekly for 4 additional weeks with variable washout period, then 1 or 3 mg/kg biweekly for 8 weeks |  | - Well-tolerated - No ADA - Transaminases decreased further from baseline - TC, LDL, TGs decreased, HDL increased | (Balwani et al., 2013)  CL-04 extension of CL-01 |
|  | 7 | Continued 1 or 3 mg/kg biweekly for up to 52 weeks |  | - One patient with severe IAR but desensitized and resumed treatment - Transaminases decreased further and normalized - LDL decreased, HDL increased further - Decreased liver fat content | (Valayannopoulos et al., 2014)  further extension of CL-04 |
|  | 7 | Continued 1 or 3 mg/kg biweekly for up to 5 years total treatment |  | - One additional patient with severe IAR but resumed treatment - One patient positive for ADA but later negative - Transaminases normalized and all maintained low levels - LDL remained low and HDL remained increased - Liver volume and liver and spleen fat content decreased | (Malinová et al., 2020)  further extension of CL-04 |
| CESD  Age 4-58 | 66, (36 treatment, 30 placebo) | 1 mg/kg biweekly 20 weeks |  | - Well-tolerated: most patients experienced mild adverse events, one serious infusion reaction which resolved with diphenhydramine - 5 patients with ADA, but did not affect safety or efficacy - Transaminases decreased, some to normal levels - Hepatic fat content reduced - LDL initially increased then LDL, TG, and apoB decreased, HDL and apoA1 increased | (Burton et al., 2015a)  double-blind period |
| CESD  Age 4-58 | 65 | 1 mg/kg biweekly 16 additional weeks (=36 total) |  | - Transaminases in placebo patients switched to sebelipase alfa dropped - LDL decreased further from baseline | (Burton et al., 2015a)  partway through open-label period |
| CESD  Age 4-58 | 65 open-label, 47 expanded | 1 mg/kg biweekly for 20 weeks, then 1 mg/kg or 3 mg/kg biweekly for an additional 130 weeks, for 104 weeks (= 256 weeks total). One patient received a few doses of 0.35 mg/kg. |  | - Most patients experienced mild-moderate adverse events, with one severe infusion reaction. - 6 patients had ADAs, 3 also had high ALTs but 5/6 later tested negative for ADAs. - Lowered transaminases sustained, many normalized - Lowered TC, LDL, and TG and raised HDL were maintained - Liver volume, liver fat content, and spleen volume reduced from baseline - Reduced steatosis | (Burton et al., 2022)  open-label and expanded open-label period |
| WD  0-2 months | 5 | 3 or 5 mg/kg weekly or biweekly for 12-116 months | One patient 5.1 nmol/h/mg (control: 31.2), one 57 umol/h/g (normal: 350-200) in leukocytes, three had 0 by DBS (nmol/punch/h) | - One patient anaphylaxis on first infusion, but continued treatment - Transaminases reduced but not statistically significant - HDL increased but did not normalize, effect on other lipids unclear - Digestive symptoms resolved in all patients, hepatosplenomegaly resolved in 4/5 - All patients survived, compared to natural history of 100% mortality | (Demaret et al., 2021) |
| WD  Age 1-6 months | 9 | 0.35 mg/kg weekly, escalated to 1, 3, or 5 mg/kg | 5-65 umol/g/h in PBMCs, 0.004-0.018 nmol/punch by DBS. Not measured in all patients. | - 5 patients had infusion reactions, three severe, but continued treatment. - Four patients developed ADAs but three later tested negative - Decreased transaminases, normalization in most patients - LDL decreased, TG decreased and normalized, HDL increased - Digestive symptoms resolved - Hepatosplenomegaly reduced - Increased weight for age centile - 67% survival (6/9) to 12 months, 56% (5/9) to 24 months, compared to 0% historical control. Patients who did not survive only received 1-4 infusions. | (Jones et al., 2017)  VITAL |
| WD  1-6 months | 9 | 3 or 5 mg/kg weekly |  | - Most patients had mild infusion reactions. - Transaminases remained low - LDL decreasing trend and HDL increased - Weight and length for age z scores increased - 56% survival to 4 years | (Vijay et al., 2021)  VITAL extension |
| WD  0.5-4 months | 10 | 1, 3, or 5 mg/kg weekly |  | - Most patients had mild infusion reactions. - 5 serious adverse events related to treatment: 4 infusion reactions, but all continued treatment. - 6 patients had ADA, 3 affected treatment efficacy - Transaminases normalized in most patients - TGs decreasing trend and HDL increased - Weight and length for age z scores increased - 79% survival to 12 months, 80% survival to 3 years | (Vijay et al., 2021)  CL-08 |
